# Hedgehog signal inhibitors suppress the invasion of human rhabdomyosarcoma cells

**DOI:** 10.1007/s00383-013-3369-6

**Published:** 2013-08-29

**Authors:** Takaharu Oue, Shuichiro Uehara, Hiroaki Yamanaka, Motonari Nomura, Noriaki Usui

**Affiliations:** Division of Pediatric Surgery, Department of Surgery, Osaka University Graduate School of Medicine, 2-2 Yamadaoka, Suita, Osaka 565-0871 Japan

**Keywords:** Rhabdomyosarcoma, Hedgehog signaling pathway, Inhibitors, Cell invasion, Cell motility, Metastasis

## Abstract

**Purpose:**

In the treatment of rhabdomyosarcoma (RMS), invasion and metastasis remain the most critical determinants of resectability and survival. The objective of this study was to determine whether Hedgehog (Hh) signaling plays a role in the invasion of RMS.

**Methods:**

Two kinds of specific Hh signaling inhibitors, cyclopamine and forskolin, were used to suppress activated Hh signals in three RMS cell lines. The effects of the Hh signaling inhibitors on tumor cell invasion and motility were investigated using Matrigel invasion assays and wound closure assays, respectively.

**Results:**

The number of invaded cells counted in six random microscopic fields in the Matrigel chambers was significantly decreased by both cyclopamine and forskolin in every RMS cell line. Furthermore, the wound closure assays revealed that a blockade of the Hh signaling pathway by the Hh inhibitors strongly impairs RMS cell motility, as visualized by the delayed closure of the gaps generated in the cultured cell monolayers of the three RMS cell lines.

**Conclusions:**

Both the invasive capacity and motility of RMS cells are significantly suppressed by Hh signaling inhibitors, demonstrating that the Hh pathway plays an important role in the invasion of RMS. Hh inhibitors may provide a new paradigm for the treatment of RMS.

## Introduction

Rhabdomyosarcoma (RMS) is the most common soft tissue sarcoma in children and constitutes approximately 9 % of pediatric solid tumors. RMS has been classified histopathologically into two major types: the embryonal-type (ERMS) and the alveolar-type (ARMS). These types are distinguished on the basis of prognosis and the degree of cell differentiation [1]. A recent study estimated the 3-year failure-free survival rate of patients with ARMS and ERMS to be 66 and 83 %, respectively [2].

The Hedgehog (Hh) pathway is a major tissue growth regulator. The pathway is activated by the binding of mammalian Hh ligands [Sonic Hh (Shh), Indian Hh and Desert Hh] to Patched (Ptch1), which inhibits Smoothened (Smo), thus culminating in the nuclear localization of DNA-binding Gli transcription factors. These transcription factors increase the production of Hh target genes, such as Ptch1 and Gli1, which serve as convenient readouts of pathway activation. Together, these components generally function to control tissue development in many tissue types [3–5]. The activation of Hh signaling has been implicated in the progression of a variety of tumors, including basal cell carcinoma of the skin, cerebellar medulloblastoma and cancers of the stomach, colon, pancreas, lungs and prostate [6–8]. Gli transcription factors are often overexpressed in these cancers and contribute to the progression of a variety of neoplasms via the regulation of cell cycle progression and apoptosis [9, 10].

To investigate the role of Hh signaling activity in pediatric malignancies, we previously detected the expression levels of Hh pathway components (Shh, Ptch1 and Gli2) in various pediatric tumors [11]. In that study, we observed that Hh signaling is broadly activated in pediatric malignancies, including RMS. Moreover, we showed that the expression levels of the Hh signaling pathway are increased in RMS cell lines [12]. Subsequently, we investigated the effects of the Hh signaling inhibitor, forskolin, an antagonist of the Gli family of transcriptional effectors, on cell proliferation and xenograft tumor growth [13]. We demonstrated that the inhibition of Hh signals suppresses tumor growth both in vitro and in vivo. These findings suggest that activation of the Hh signaling pathway contributes to the development of RMS. Therefore, the Hh signaling pathway could be a therapeutic target for the treatment of RMS.

Recent studies have suggested that the Hh pathway plays an important role, not only in tumor growth, but also in metastatic behavior. Studies conducted by Hochman et al. [14] have suggested that the components of the Shh signaling pathway participate directly in cell migration and angiogenesis, and inhibition of this pathway blocks Shh-induced cell migration and angiogenesis. The relationship between the Hh signaling pathway and tumor invasion and metastasis has been investigated in various adult cancers, including pancreatic cancer, gastric cancer, hepatocellular carcinoma and bladder carcinoma [15–19]. The results of these studies imply that increased Hh signaling contributes to the maintenance of metastatic behavior. Therefore, understanding the pathways involved in tumor metastasis will provide great promise for the discovery of novel therapeutics and the treatment of invading and metastatic diseases. However, to date, there is no evidence of any relationship between Hh signaling and tumor invasiveness or metastasis in pediatric malignancies. In this study, we investigated the effects of Hh signaling inhibitors on RMS cell migration activity to determine whether Hh signaling plays a role in RMS invasion and metastasis.

## Methods

### Cell cultures

Three cell lines derived from pediatric RMS, RMS-YM (ERMS), RD (ERMS) and RH30 (ARMS), were used in these studies. *PAX3*-*FKHR* fusion gene was detected in RH30 cells derived from ARMS (data not shown). All of the cell lines were routinely maintained at 37 °C and 5 % CO2 in Dulbecco’s modified essential medium (DMEM) supplemented with 10 % fetal bovine serum (FBS) and 1 % penicillin/streptomycin.

### Matrigel invasion assays

**Fig. 1:**
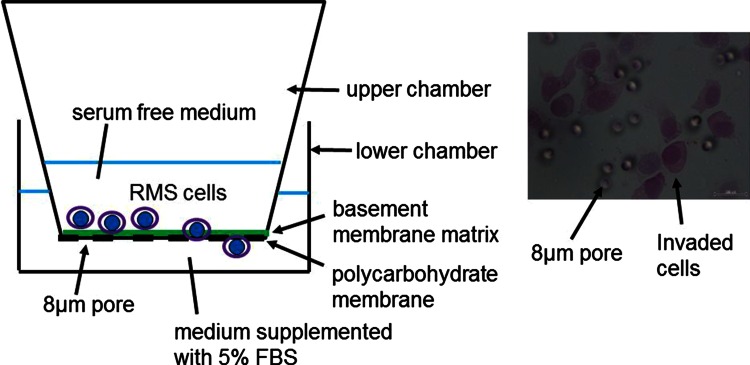
Principle of the Matrigel invasion assay The Matrigel invasion chamber has an 8 μm pore size polycarbohydrate membrane and the upper surface of the membrane is coated with a uniform basement membrane matrix (BMM). The chambers were placed into the lower chambers filled with the medium supplemented with 5 % FBS as a chemoattractant, therefore cells will invade into BMM and move to the lower surface of the membrane through the 8 μm pores. After a 22-h incubation, nonmigratory cells from the upper surface of the filter were removed and invasive cells that had passed through to the lower surface of the filter were fixed and stained

Cell invasion was evaluated using a BioCoat Matrigel invasion chamber (BD Bioscience, Bedford, MA, USA) (Fig. 1). Cell suspensions (5 × 10^4^ cells/ml) of RMS-YM, RD and RH30 cells were prepared in serum-free culture medium in the absence (control) or presence of the Hh inhibitors, cyclopamine (10 μM; Sigma Aldrich Co., Tokyo, Japan) or forskolin (100 μM; Sigma Aldrich Co., Tokyo, Japan). 500 μl of each cell suspension was added to the Matrigel invasion chamber. The chambers have an 8 μm pore size polycarbohydrate membrane and the upper surface of the membrane is coated with a uniform basement membrane matrix (BMM). The upper chambers were placed into the lower chambers, which were filled with 750 ml of DMEM supplemented with 5 % FBS as a chemoattractant so that the cells would invade the BMM and move toward the lower surface of the membrane through the 8 μm pores. After 22 h of incubation in a tissue culture incubator at 37 °C, nonmigratory cells from the upper surface of the filter were removed and invasive cells that had passed through to the lower surface of the filter were fixed and stained. The number of invading cells in six random fields was counted using bright field microscopy at 200× magnification. The experiments were performed three times using duplicate samples.

### Wound closure assays

For the scratch wound closure assays, freshly confluent monolayers of s RMS-YM, RD and RH30 cells were wounded by manual scraping with a sterile pipette tip. Following wounding of the monolayers, wound sizes were verified to ensure that they were all the same width (approximately 0.8 mm). In the Hh inhibition groups, the cell culture medium was replaced with fresh culture medium containing cyclopamine (10 μM) or forskolin (100 μM). Wound closure was monitored over a 48-h period with a phase contrast microscope at 200× magnification. The migration rates were assessed as the percentage of wound closure by measuring the distance between the wound edges at time intervals of 4 h until the wounds were completely closed. The experiments were repeated three times in all groups.

### Statistical analysis

All of the experiments were independently performed at least three times, and the data were represented as the mean with the standard deviation for each parameter. The statistical analyses were performed using unpaired Student’s *t* test, and *P* values <0.05 were considered to be statistically significant.

## Results

### Matrigel invasion assays

**Fig. 2:**
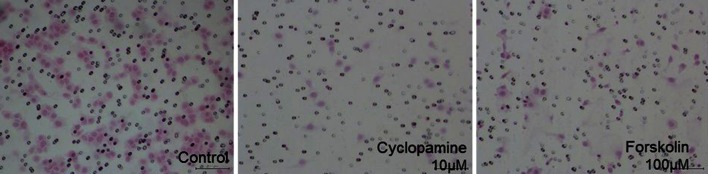
Matrigel invasion assays RMS-YM, RH and RH30 cells were seeded onto a Matrigel invasion chamber containing either cyclopamine (10 μM) or forskolin (100 μm) for the cell invasion assay and incubated for 22 h. The cells that actively migrated to the lower surface of the filters were stained and counted using bright field microscopy at ×200 in 6 random fields. The photographs show the typical staining of the RMS-YM cells in the control group, cyclopamine (10 μM) treated group and forskolin (100 μm) treated group

**Fig. 3:**
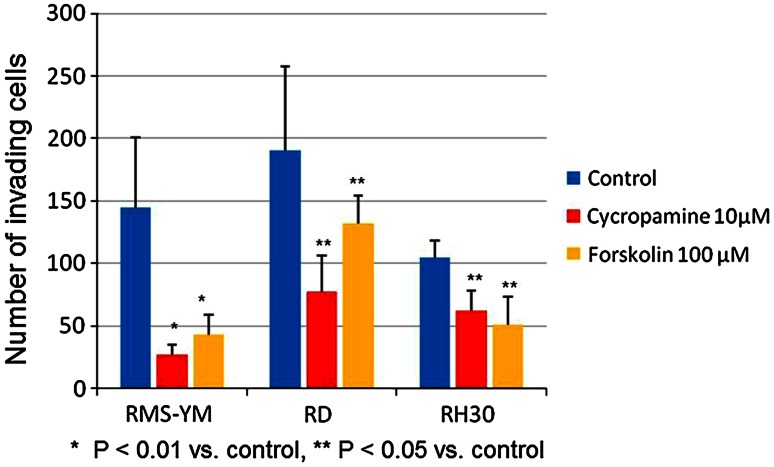
Number of invading cells in the Matrigel invasion assays The number of invading cells significantly decreased in both cyclopamine and forskolin-treated groups compared with the control groups in the three RMS cell lines

We employed cyclopamine and forskolin (specific inhibitors of the Hedgehog pathway) to block the Hh pathway in the RMS cell lines and then assessed the changes in the invasive potential of the cells. The Matrigel invasion assays indicated that RD cells exhibit the strongest invasive potential. As shown in Figs. 2 and 3, the number of invaded cells counted in six random microscopic fields in the Matrigel chamber was significantly decreased by both cyclopamine and forskolin in every RMS cell line. The mean invasiveness for the control, cyclopamine-treated and forskolin-treated RMS-YM cells was 145.2 + 55.5, 27.2 + 7.9 and 43.0 + 16.3 cells (*P* < 0.01)/6 random microscopic fields, respectively. The mean invasiveness for the control, cyclopamine-treated and forskolin-treated RD cells was 190.7 + 67.2, 77.3 + 29.0 and 131.5 + 22.7 cells (*P* < 0.05)/6 random microscopic fields, respectively. The mean invasiveness for the control, cyclopamine-treated and forskolin-treated RH30 cells was 104.3 + 14.1, 62.2 + 16.0 and 51.3 + 21.9 cells (*P* < 0.05)/6 random microscopic fields, respectively.

### Wound closure assays

**Fig. 4:**
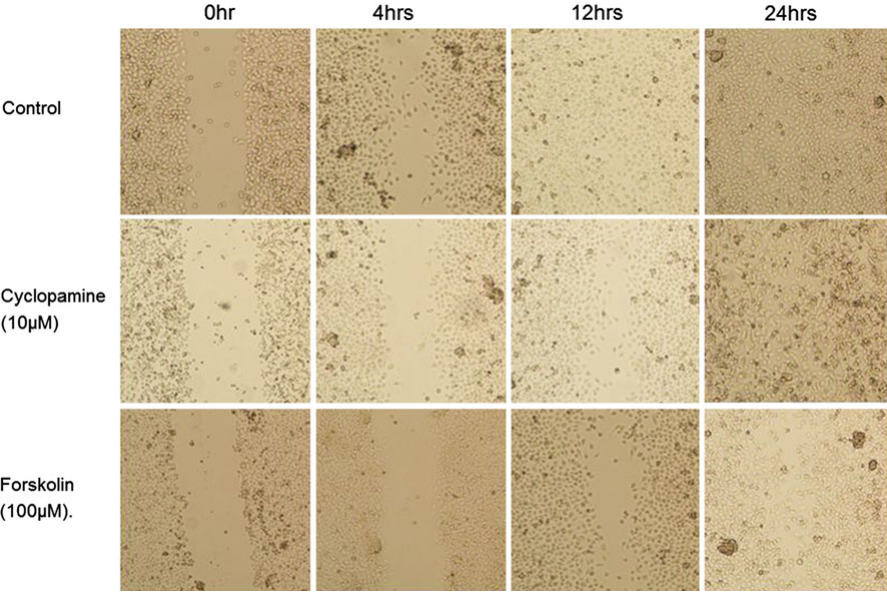
Wound closure assays. Confluent monolayers of RMS-YM, RH and RH30 cells were wounded by scratching with a pipette tip. The culture medium was replaced with fresh medium containing either cyclopamine (10 μM) or forskolin (100 μM) to suppress Hh signals. Wound closure was monitored using phase contrast microscopy. Photos were taken immediately (0 h) and every 4 h until the wounds closed. The experiments were repeated three times with similar results. A representative photomicrograph of RH-30 cells in each condition at ×200 magnification is shown. In the control group, the wound was closed at 12 h, whereas in the cyclopamine or forskolin-treated group, the wound was closed at 24 h or later

**Fig. 5:**
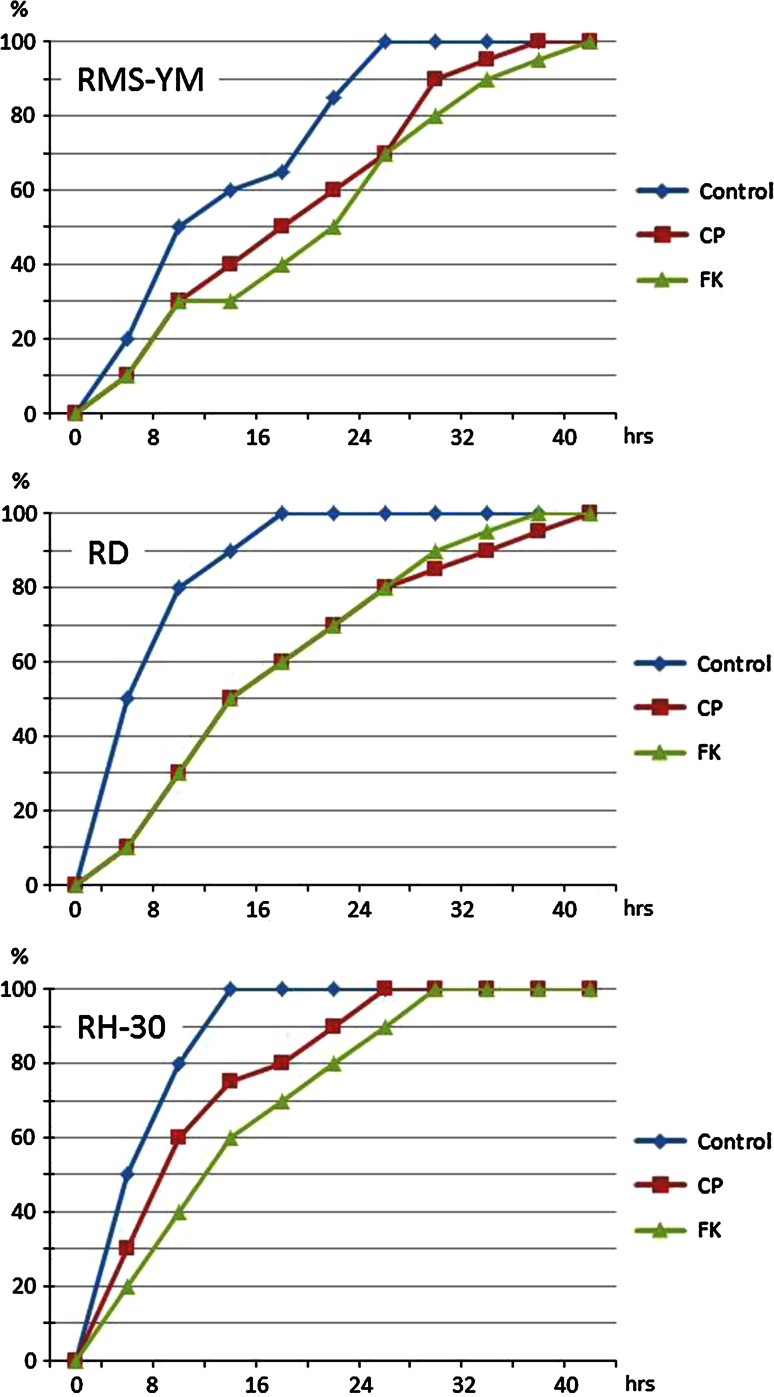
Percentage of wound closure in the monolayer of each RMS cells at every 4 h. Time to wound closure for the cyclopamine-treated and forskolin-treated RMS cells was significantly delayed compared with that of the non-treated control cells. The blockade of the Hh signaling pathway by time to closure of the control, cyclopamine-treated and forskolin-treated RMS-YM cells was 24, 36 and 40 h; RD cells was 16, 40 and 36 h, and RH30 cells was 12, 24 and 28 h, respectively

The wound closure assays revealed that the Hh inhibitors strongly impair RMS cell motility, as visualized by the delayed closure of the gaps generated in cultured cell monolayers of the three RMS cell lines (Fig. 4). Time to wound closure for the cyclopamine-treated and forskolin-treated RMS cells was significantly delayed compared with that of the non-treated control cells. The blockade of the Hh signaling pathway by time to closure of the control, cyclopamine-treated and forskolin-treated RMS-YM cells was 24, 36 and 40 h, respectively. The blockade of the Hh signaling pathway by time to closure of the control, cyclopamine-treated and forskolin-treated RD cells was 16, 40 and 36 h, respectively. The blockade of the Hh signaling pathway by time to closure of the control, cyclopamine-treated and forskolin-treated RH30 cells was 12, 24 and 28 h, respectively (Fig. 5).

## Discussion

Abundant evidence indicates that Shh signaling is involved in tumorigenesis [3–13]. Although some studies, including ours, have suggested that overexpression of Hh signaling correlates with tumor proliferation in RMS [12, 13], no studies have so far described the role of Hh signaling in the invasive or metastatic progression of RMS. To our knowledge, this is the first report to specifically show a relationship between Hh signaling and cell invasion in RMS.

In this study, we investigated the functional role of Hh signaling in the invasiveness of RMS cells in vitro using two types of Hh signaling inhibitors, cyclopamine and forskolin. Cyclopamine, which inhibits the Hh pathway by antagonizing Smo [20, 21], is reported to suppress the growth of malignant tumors, such as pancreatic tumors and gastric tumors [7, 8, 16]. Forskolin is an antagonist of the Gli family of transcriptional effectors, which form proteins in the final stage of the Hh signaling pathway. Gli1 is considered to be a marker for the activation of the Hh signaling pathway [5, 10], and forskolin is considered to suppress the Hh signaling pathway by suppressing Gli1. In our previous study, we treated RMS cell lines with forskolin. The results of that study revealed that Hh signaling is essential for the proliferation of RMS cells. In this study, we found that both Hh inhibitors significantly suppress the ability of RMS cells to invade through the Matrigel chamber compared to control cells. In addition, both Hh inhibitors were found to delay wound closure in the RMS cell monolayers. Collectively, these studies demonstrate that the Hh pathway might play a facilitatory role in the invasion of RMS cells.

In this study, we used two cell lines (RMS-YM, RD) established from embryonal RMS and one cell line (RH30) established from alveolar RMS. Alveolar RMS is clinically more aggressive than embryonal RMS. Although the Matrigel invasion assays have shown that there was no significant difference in the number of invading cells among three cell lines, wound closure assays have shown that time to wound closure for the RH 30 cells was shorter (12 h) than that of the RMS-YM(24 h) and RM cells (16 h). These findings may indicate that motility of RMS cells is higher in alveolar RMS than in embryonal RMS, reflecting the biologic aggressiveness of alveolar rhabdomyosarcoma. Inhibition of Hh signaling reduced the invasive capacity and motility of both embryonal and alveolar RMS cells, therefore Hh signaling inhibitors may be effective to suppress the invasiveness of both embryonal and alveolar RMS.

Although the mechanistic processes underlying the effects of Hh signals on cell invasion currently remain uncertain, there exist some relevant reports on adult cancers. Feldmann et al. [15] has reported that, in pancreatic cancer cell lines, Hh inhibition by cyclopamine results in the downregulation of snail and the upregulation of E-cadherin, consistent with the inhibition of epithelial-to-mesenchymal transitions, which is mirrored by a striking reduction in in vitro invasive capacity. Conversely, Gli1 overexpression in immortalized human pancreatic ductal epithelial cells leads to the development of a markedly invasive phenotype and the near total downregulation of E-cadherin. Yoo et al. [16] has reported that, in mouse xenograft models of human gastric cancer, the enforced expression of Shh significantly enhances the incidence of lung metastases compared with non-expressing controls. Moreover, these authors revealed that Shh signaling promotes the metastasis of gastric cancer through the activation of the PI3 K/Akt pathway, which leads to mesenchymal transition and matrix metalloproteinase 9 activation. Further investigations are needed to elucidate the regulatory mechanisms underlying Hh signaling in the invasion and metastatic progression of RMS cells.

Although treatment of RMS has dramatically improved over the past 20 years, the prognosis of patients with high-risk tumors remains poor due to a high rate of recurrence and metastasis. Therefore, the development of new treatment strategies is essential. Surgical resection is an important therapeutic option; however, many patients develop local recurrence and/or distant metastases following curative surgical resection and postoperative chemotherapy. Local recurrence often occurs at the tumor site even after complete surgical resection, and most cases of recurrence occur in the first 2 years. Early recurrence is considered to be the result of residual microscopic foci of malignant cells that are undetectable at the time of surgery.

There is a significant need to target the pathways responsible for RMS recurrence and to develop therapies specifically aimed at preventing or delaying RMS recurrence and metastasis. Our results indicate that Hh signaling inhibitors suppress tumor cell invasiveness and motility, suggesting that these inhibitors can reduce the incidence of local recurrence by suppressing tumor invasion in the original site when used prior to surgery. Moreover, distant metastases are also controlled by preventing tumor cell invasion into vessels. Therefore, the use of Hh inhibitors may provide a new paradigm for the treatment of disseminated RMS, particularly when used in combination with conventional chemotherapy and surgical resection.

Currently, there is no available clinical treatment specifically targeting the Hh signaling pathway. In recent years, derivatives of cyclopamine and other small molecular antagonists that target Hh signaling have entered clinical phase I/phase II trials [22, 23]. Treatments using these compounds are expected to be clinically available in the near future. We hope that these developments will be effective in treating children with RMS.
